# Anti-MDA5 Antibodies in a Large Mediterranean Population of Adults with Dermatomyositis

**DOI:** 10.1155/2014/290797

**Published:** 2014-02-04

**Authors:** Moises Labrador-Horrillo, Maria Angeles Martinez, Albert Selva-O'Callaghan, Ernesto Trallero-Araguas, Eva Balada, Miquel Vilardell-Tarres, Cándido Juárez

**Affiliations:** ^1^Internal Medicine Department, Vall D'Hebron General Hospital, Universitat Autonoma de Barcelona, Passeig Vall D'Hebron 119-129, 08035 Barcelona, Spain; ^2^Immunology Department, Hospital de La Santa Creu I Sant Pau, Universitat Autonoma de Barcelona, Barcelona, Spain

## Abstract

A new myositis-specific autoantibody directed against melanoma differentiation-associated gene 5 (anti-MDA5) has been described in patients with dermatomyositis (DM). We report the clinical characteristics of patients with anti-MDA5 in a large Mediterranean cohort of DM patients from a single center, and analyze the feasibility of detecting this autoantibody in patient sera using new assays with commercially available recombinant MDA5. The study included 117 white adult patients with DM, 15 (13%) of them classified as clinically amyopathic dermatomyositis (CADM). Clinical manifestations were analyzed, with special focus on interstitial lung disease and its severity. Determination of anti-MDA5 antibodies was performed by a new ELISA and immunoblot technique. In sera, from 14 (12%) DM patients (8 CADM), MDA5 was recognized by ELISA, and confirmed by immunoblot. Eight of the 14 anti-MDA5-positive patients (57.14%) presented rapidly-progressive interstitial lung disease (RP-ILD) versus 3 of 103 anti-MDA5-negative patients (2.91%) (*P* < 0.05; OR: 44.4, 95% CI 9.3–212). The cumulative survival rate was significantly lower in anti-MDA5-positive patients than in the remainder of the series (*P* < 0.05). Patients with anti-MDA5-associated ILD presented significantly lower 70-month cumulative survival than antisynthetase-associated ILD patients. Among the cutaneous manifestations, only panniculitis was significantly associated with the presence of anti-MDA5 antibodies (*P* < 0.05; OR: 3.85, 95% CI 1.11–13.27). These findings support the reliability of using commercially available recombinant MDA5 for detecting anti-MDA5 antibodies and confirm the association of these antibodies with RP-ILD in a large series of Mediterranean patients with DM.

## 1. Introduction

In 2005, Sato et al. [1] identified a novel autoantibody recognizing a 140-kDa protein in patients with dermatomyositis (DM), particularly in those with clinically amyopathic dermatomyositis (CADM). The 140-kDa autoantigen, which was identified as melanoma differentiation-associated protein 5 (MDA5), is detected in 19% to 35% of the patients with DM. In the Asian population, this autoantibody seems to be associated with rapidly progressive interstitial lung disease and with severe cutaneous vasculopathy (skin ulceration, tender palmar papules, or both) [1–6]. Recently, the presence of anti-MDA5 antibody-associated dermatopulmonary syndrome was described in the white population [7–9].

MDA5, also known as interferon-induced helicase-1 (IFIH1), is a member of the retinoic acid-inducible gene I-like helicase (RIG-I or RLH) family of proteins, [10] which function by recognizing single-stranded RNA viruses and are involved in the innate immune response, including type I IFN production [11].

The main drawback to routine use of this antibody for clinical purposes is that its determination is limited to techniques that are only available in research laboratories, such as immunoprecipitation of radioactive-labeled protein [8] or enzyme-linked immunoassay (ELISA) using in-house fabricated recombinant proteins [12, 13].

Our objective was to evaluate the prevalence and clinical manifestations of anti-MDA5-positive patients in a large cohort of DM patients from a single center in Barcelona, and to determine the feasibility of detecting this autoantibody with the use of more widely available techniques (ELISA and immunoblotting) with commercially available recombinant MDA5 as the antigen.

## 2. Patients and Methods

### 2.1. Patient Population

This study was performed in 117 adult patients (92 women) with DM (15 with clinically amyopathic DM). In addition, 45 patients with polymyositis (PM), 30 with systemic sclerosis (SSc), and 25 with systemic lupus erythematosus (SLE) were included as controls. Twenty-five healthy controls were also included to determine the cut-off value for establishing the positive status by ELISA. Healthy and disease controls were age and sex matched to the DM patients. The median age of DM patients was 52 years (range 22–81). The patients studied belong to a historical cohort diagnosed with idiopathic inflammatory myopathy at Vall d'Hebron General Hospital in Barcelona (Spain), between 1983 and 2012. Our center is a single teaching hospital with approximately 700 acute care beds, attending a population of nearly 450,000 inhabitants. All myositis patients in this population are referred to our hospital for diagnosis and therapy, regardless of the severity of the disease. Serum samples from these patients are routinely collected at diagnosis and during follow-up in our outpatient clinic, and stored at −80°C. Patients and controls included in the study gave informed consent for the use of their serum for research purposes. The study was approved by the institutional review board of our hospital.

The diagnosis of DM and PM was based on the criteria of Bohan and Peter [14, 15]. Only patients with definite or probable disease were included. The Sontheimer criteria were used to diagnose amyopathic DM [16]. Interstitial lung disease (ILD) was diagnosed according to the consensus classification of idiopathic interstitial pneumonias. The diagnosis of ILD was established by high-resolution CT findings, and rapidly progressive-interstitial lung disease (RP-ILD) was defined as a worsening of radiologic interstitial changes with progressive dyspnea and hypoxemia within 1 month after the onset of respiratory symptoms. [17] Cancer-associated myositis (CAM) was defined as cancer occurring within 3 years of the myositis diagnosis. Patients received treatment with corticosteroids and immunosuppressive drugs (methotrexate, azathioprine, calcineurin inhibitors [cyclosporine A or tacrolimus], or cyclophosphamide pulses) were added when needed; intravenous immunoglobulin was used as an adjuvant therapy, and biological therapy (rituximab) was also instituted when possible in refractory cases. A drug trial was defined as a single course from the beginning of the administration of a given drug to the time at which the drug was discontinued, or in the case of prednisone, as the time at which the dose was reduced to one quarter of the initial dose. Clinical data were obtained retrospectively by review of the patients' medical records.

### 2.2. Laboratory Tests and Serological Assay

Serum samples from each patient were screened by indirect immunofluorescence for antinuclear antibodies (ANA) using HEp-2 cells, and by a commercial ELISA used in our routine laboratory setting for antibodies against extractable nuclear antigens (Ro, La, RNP, Sm) and anti-histidyl-tRNA synthetase (anti-Jo-1). Anti-TIF1*γ* antibodies were detected by an in-house ELISA and confirmed by immunoblot [18]. In addition, all samples were tested by protein and RNA immunoprecipitation [19], which enabled detection of other synthetases and myositis-specific and myositis-associated antibodies (anti-Mi-2, anti-SRP, anti-Ro52, anti-Ro60, anti-La, anti-PM/Scl, anti-p155, and anti-U1RNP) that may have been overlooked by ELISA, and confirmed the ELISA results.

### 2.3. Anti-MDA5 ELISA

Briefly, 96-well ELISA plates (NUNC, Kamstrup, Denmark) were coated with 100 ng of purified recombinant MDA5 (OriGene, Rockville, MD), diluted in phosphate buffered saline (PBS), and left to stand overnight at 4°C. Wells were incubated for 1 hour at room temperature (RT) with blocking buffer (10% nonfat dry milk in PBS). Plates were then washed (HRP Wash, INOVA Diagnostic Inc., San Diego, CA); human serum samples diluted 1 : 100 in blocking buffer were added in triplicate: two to MDA5-coated wells and one to a PBS-coated well (without antigen) to determine the background absorbance. Plates were incubated at RT for 1 hour. After washing, HRP-labeled goat anti-human IgG antibody (INOVA Diagnostic Inc., San Diego, CA) was added to each well, and plates were incubated for 1 h at RT and washed again. Color development was performed with peroxidase reagent TMB Chromogen (INOVA Diagnostic Inc., San Diego, CA) and absorbances at 450 nm were determined. For each sample, the background absorbance from the PBS-coated well was subtracted from that of the corresponding MDA5-coated wells (the average of the two results). Sample absorbance was expressed as optical density units. The same positive serum (from patient 11, confirmed by IP by Casciola Rosen, from Baltimore, USA) was used as the reference in each assay.

### 2.4. Anti-MDA5 Immunoblot

**Figure 1:**
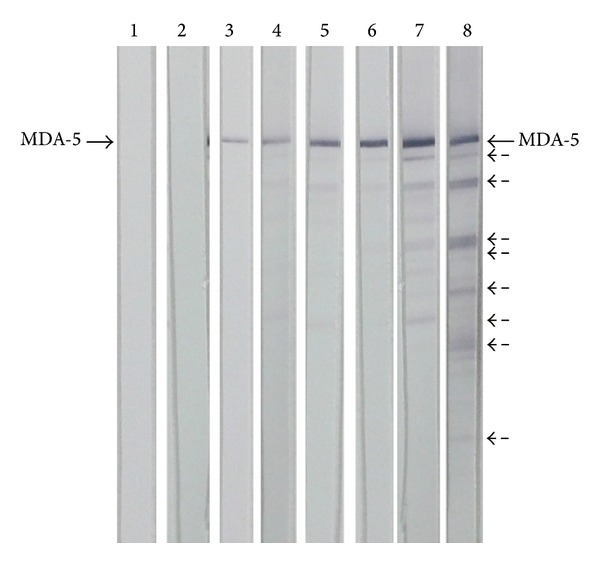
Immunoblots showing the reactivity of IgG antibodies from dermatomyositis patients against commercially available purified recombinant MDA5. Lanes 3 and 4 (+), 5 and 6 (++), and 7 and 8 (+++) were considered positive results. Lanes 1 and 2 corresponded to negative serum samples. Dashed arrows are probably degradation products of MDA5.

Briefly, 5 *μ*g of purified recombinant MDA5 (OriGene, Rockville, MD) was run on 4% to 12% polyacrylamide-SDS minigels with MOPS running buffer, and western blot was performed on a nitrocellulose membrane using the Invitrogen NuPAGE (Carlsbad, CA) electrophoresis system. [20] MDA5-transferred nitrocellulose was vertically cut into several strips and incubated for 1 hour at RT in PBS with 0.05% Tween (PBS-T) containing 3% nonfat dry milk (blocking buffer). Each strip was then incubated with the corresponding human serum sample diluted 1 : 100 in blocking buffer for 1 hour at RT. After washing, phosphatase alkaline-labeled goat anti-human IgG antibody (Dako, Glostrup, Denmark 1 : 2000) was added to each strip and strips were incubated for 1 hour at RT. Color development was performed by phosphatase reagent (BCIP/NBT, Sigma-Aldrich, St. Louis, MO). Based on signal intensity, the results were classified into negative, weak positive (+), or positive (++, +++) (Figure 1).

### 2.5. Statistical Analysis

Associations between anti-MDA5 antibodies and qualitative variables were evaluated with the chi-square and Fisher exact test. The strength of the associations between variables was measured using odds ratios (ORs) with 95% confidence intervals (CIs). The Mann-Whitney *U* test was used for comparisons of median values. The corresponding area under the curve (AUC) of the ROC analysis of anti-MDA5 antibody for detection of RP-ILD, CADM, and total DM was analyzed with 95% of CIs. All tests were two-sided, and probability (*P*) values of <0.05 were considered statistically significant. Cumulative survival rates were estimated by the Kaplan-Meier test. The log-rank test was also used to compare survival rates. All analyses were performed with SPSS, version 19.0 (SPSS, Chicago, IL).

## 3. Results

**Figure 2:**
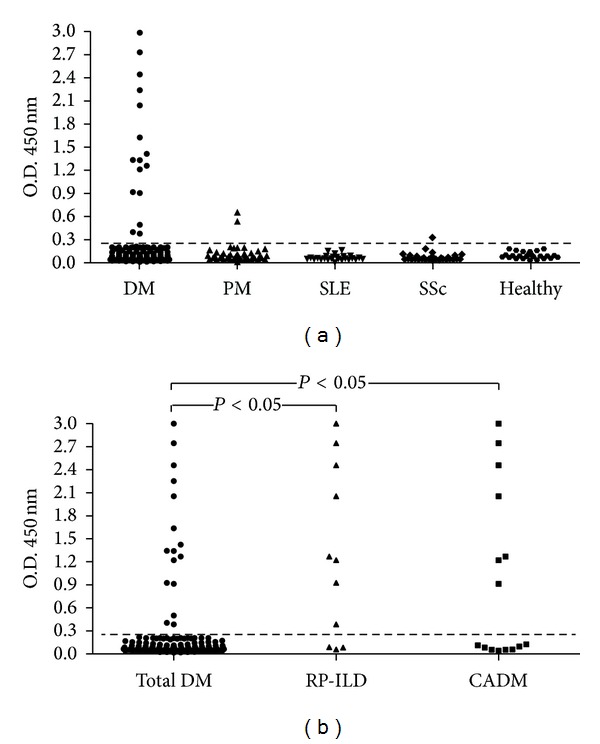
Representation of the anti-MDA5 ELISA test results in patients with dermatomyositis (DM) (*n* = 117) and controls groups: polymyositis (PM) (*n* = 45), systemic sclerosis (SSc) (*n* = 30), systemic lupus erythematosus (SLE) (*n* = 25), and healthy controls (*n* = 25). Panel (b) shows anti-MDA5 ELISA of patients with DM (*n* = 117) and individual subgroups of DM patients: rapidly progressive-interstitial lung disease (RP-ILD) (*n* = 11) and clinically amyopathic dermatomyositis (CADM) (*n* = 15). The cut-off value for a positive result was established at 0.188 absorbance units which corresponded to 2 standard deviations above the mean value obtained for the 25 healthy controls (dashed line).

One-hundred and seventeen adult DM patients, 15 of whom had CADM, were included in the study. Anti-MDA5 was determined by our in-house ELISA and immunoblot techniques using a commercially available recombinant MDA5. The cut-off value for a positive result on ELISA was established at 0.188 absorbance units, which corresponded to 2 standard deviations above the mean value obtained for the 25 healthy controls. The other control subjects included 45 patients with polymyositis (PM), 30 with systemic sclerosis (SSc), and 25 with systemic lupus erythematosus (SLE) (Figure 2). Only two patients diagnosed with DM, 2 with PM, and 1 with SSc showed weak anti-MDA5 reactivity by ELISA, which was not confirmed by immunoblot; these results were considered false positives. Anti-MDA5 antibodies detected by ELISA and confirmed by immunoblot were only found in DM patients. ROC curve analysis for the positivity of anti-MDA5 of all patients with DM against controls and CADM patients versus remaining DM disclosed an AUC of 0.56, 95% CI 0.49–0.63 and 0.74, 95% CI 0.58–0.9, respectively. Patients with the highest absorbance unit values on ELISA also showed the strongest anti-MDA5 positivity on immunoblotting.

Fourteen patients, 8 with CADM, tested positive for anti-MDA5, which represents a prevalence of 12% of the DM patients from our cohort. Median (range) age at diagnosis of anti-MDA5 positive patients was 47 (28–60) years, which did not differ significantly from the remainder of the cohort. ANA was positive in 5 patients. Seven patients also tested positive to anti-Ro52, but none of them was positive for any antisynthetase antibody. Relevant clinical and immunological findings are summarized in Table 1.

### 3.1. Relationship between Anti-MDA5 and RP-ILD

Interstitial lung disease was present in 9 of the 14 (64.3%) patients with anti-MDA5 autoantibodies, and the condition was rapidly progressive in 8 patients. RP-ILD was more frequent in patients with CADM, both in the anti-MDA5 positive group (7 of 8 CADM versus 1 of 6 DM; *P* < 0.05) and in the overall cohort (8 of 15 CADM patients versus 3 of 102 DM patients; *P* < 0.05). When RP-ILD was evaluated in relation to anti-MDA5 positivity, a highly significant association was observed between the two parameters. Thus, 8 of the 14 anti-MDA5-positive patients presented RP-ILD versus 3 of the 103 anti-MDA5-negative patients (*P* < 0.05; OR: 44.4, 95% CI 9.3–212; AUC 0.84, 95% CI 0.68–1). Nevertheless, no association was found between anti-MDA5 ELISA titers at diagnosis of DM and development of a RP-ILD. Moreover, 6 of the 8 (75%) patients with anti-MDA5 and RP-ILD were Ro52-positive in comparison to only 1 of the 6 (16%) patients without RP-ILD.

### 3.2. Relationship between Anti-MDA5 and Cutaneous Manifestations and Cancer

The CADM diagnosis was significantly associated with anti-MDA5 positive status (8 of 14 MDA5-positive patients versus 7 of 103 MDA5-negative (*P* < 0.05; OR: 18.3, 95% CI 4.9–67.6)). No differences were observed in the frequency of Gottron's papules, heliotrope rash, photosensitivity, shawl or “V” sign, cuticular overgrowth, calcinosis, or the presence of mechanic's hands when the anti-MDA5-positive and anti-MDA5-negative groups were compared. Panniculitis was the only manifestation significantly associated with the presence of anti-MDA5 (5 out of 14 anti-MDA5-positive versus 13 out of 103 anti-MDA5-negative; *P* < 0.05; OR: 3.85, 95% CI 1.11–13.27); nevertheless, a multivariate analysis was not possible to be performed due to methodological reasons.

Cancer was diagnosed in 4 out of 14 (28.6%) patients with anti-MDA5 autoantibodies, and 3 (21%) of them fulfilled criteria of CAM. However, no association was found between anti-MDA5 autoantibodies and CAM (*P* > 0.05). A similar result was obtained when we repeated the analysis after excluding from the cohort the anti-TIF1*γ*-positive patients (28 patients) in order to avoid a possible confounding effect of the presence of these patients in the control group.

### 3.3. Survival Rates of Anti-MDA5-Positive Patients

**Figure 3:**
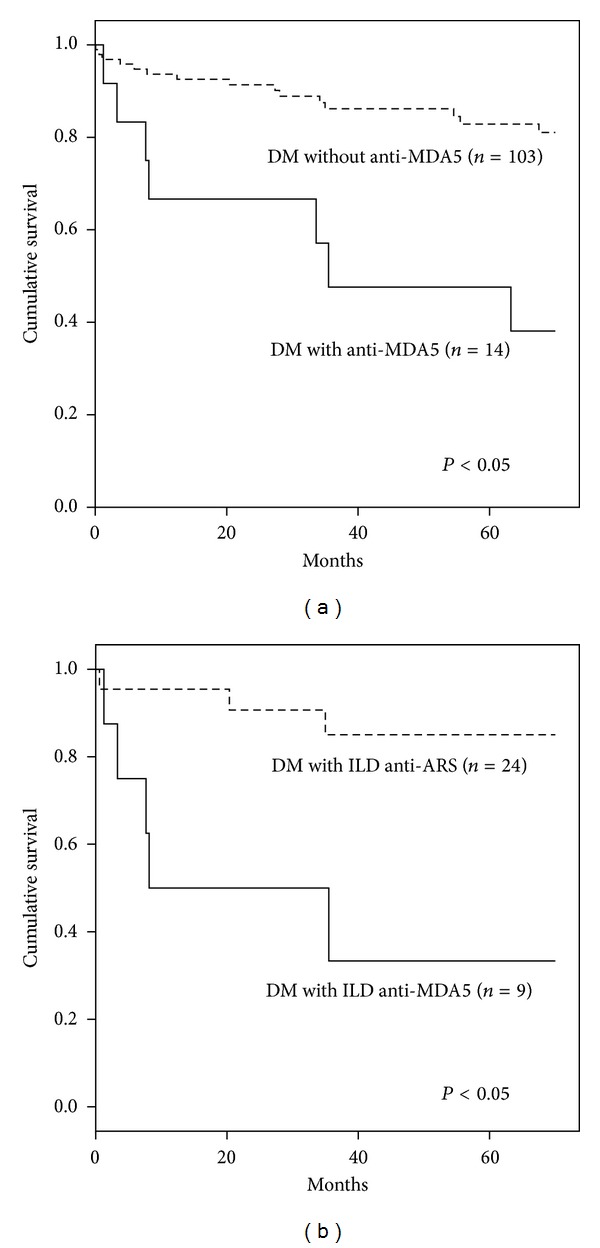
Cumulative 70-month survival rates for DM patients with and without anti-MDA5 antibody (a) and for DM patients with ILD associated with anti-ARS or anti-MDA5 (b). The 70-month cumulative survival rates were calculated using the Kaplan-Meier test. The log-rank test was also used to compare survival rates. ARS: aminoacyl-tRNA synthetase; DM: dermatomyositis; ILD: interstitial lung disease.

The cumulative 70-month survival rate was significantly lower (38%) in the group of patients with anti-MDA5 than in the remainder of the cohort (62%) (log-rank test, *P* < 0.05) (Figure 3(a)). Comparison of cumulative 70-month survival between anti-MDA5-associated ILD and antisynthetase-associated ILD also showed a statistical difference (log-rank test, *P* < 0.05) (Figure 3(b)). No differences in 70-month survival were observed between the CADM group and the classic DM group in anti-MDA5- positive patients. All groups were comparable in age, gender, and number of immunosuppressive agents added to the corticosteroid treatment. Differences in survival could not be attributed to a higher proportion of “deaths directly related to cancer” between groups.

## 4. Discussion

The results of this study prove the feasibility of detecting antibodies against MDA5 in adult patients with DM by in-house ELISA and immunoblot techniques using commercially available recombinant MDA5 as the antigen. In addition, the findings in our patients contribute to support the previously reported association of anti-MDA5 antibody with RP-ILD and CADM. This association has been mainly described in Asian patients. [1–6], and the only studies performed in a white population have come from the United States [7–9]. Our results in a large series of Mediterranean patients from a single reference center, together with those from other articles published on this topic, [1–8] indicate that anti-MDA5 antibodies may be a hallmark of adult CADM patients with RP-ILD regardless of their origin.

Furthermore, some authors have suggested an association between this autoantibody and a specific, severe skin vasculopathy in adult DM, characterized by vascular fibrin deposition with variable perivascular inflammation [3, 7, 8]. We found an association only with panniculitis, one of the mucocutaneous findings previously described by Fiorentino et al. [8] in white adults with DM and anti-MDA5 antibody.

Although we routinely test all serum samples from patients with inflammatory myopathies by immunoprecipitation assays using ^35^S-protein-labeled HeLa cells, until the development of our proposed method, we had not been able to clearly identify patients with anti-140 kDa antibodies (anti-CADM-140; i.e, anti-MDA5). In our analyses, this polypeptide migrated to an area in which almost 70% of sera, including those from normal subjects, immunoprecipitated a weak line around 140 kDa. Hence, until anti-MDA5 was described by Sato et al. [1, 6], and we tested these patients with a commercially available recombinant MDA5 by our in-house ELISA and immunoblot techniques, we were unable to recognize this autoantibody. Our positive patients correspond to a period of 30 years of follow-up. Our first patient with CADM and RP-ILD died nearly 20 years ago, and anti-MDA5 was detected in a stored frozen serum sample by our in-house techniques. Thus, these methods could represent a significant advancement in identification of this autoantibody, even in laboratories with standard equipment.

Anti-Ro52 antibodies were present in most of our patients with RP-ILD and anti-MDA5, an association that has only recently been reported in anti-MDA5-positive ILD patients [9]. This fact confers relevant significance on anti-Ro52 as a costimulatory autoantibody, a concept reported in patients with antisynthetase syndrome [21, 22].

One patient was positive to both anti-MDA5 and anti-TIF1*γ*. To our knowledge, this is the first description of this association. Coexistence of two different myositis-specific antibodies has been rarely reported [19, 23]. Hence, the situation of this patient is intriguing and warrants further investigation.

The clinical course of anti-MDA5-positive DM patients can be divided into three groups. First (and most important from the prognostic perspective), is the group of patients with CADM and RP-ILD, who usually have a poor prognosis and a mortality rate of nearly 50% despite aggressive immunosuppressive therapy and even lung transplantation. Second, the group with CADM and little lung involvement, who show skin manifestations, such as ulcerations, palmar pustules, and perhaps panniculitis, as was reported here. The prognosis does not seem to be unfavorable in this group. And finally, the third group of patients, who have ILD that is not rapidly progressive and shows a disease pattern similar to that of classic antisynthetase syndrome. [9] Differences in the prognosis between the first and the third group may be due to genetic background, and either early immunosuppressive therapy or use of certain drugs, such as calcineurin inhibitors [24].

The existence of a clinical syndrome of rapidly progressive ILD (usually in antisynthetase-negative patients) was recognized some years ago [25], but the absence of useful biomarkers made it difficult to characterize these patients. The discovery of anti-MDA5 antibodies will help to better define this population, facilitate an early diagnosis, establish the prognosis, and ultimately enable the development of randomized clinical trials to determine the optimal therapy in anti-MDA5-positive patients with a poor prognosis. Moreover, as it has been recently described, anti-MDA5 antibody measurement seems to be useful for monitoring disease activity [13, 26].

As our results show, ELISA confirmed by immunoblot with commercially available recombinant MDA5 antigen are useful techniques for anti-MDA5 detection that can be reliably performed in a standard laboratory setting, with potential application in clinical practice.

## Figures and Tables

**Table 1:** Clinical and immunological characteristics of our 14 MDA5-positive Mediterranean patients.

ID/ sex	Data onset Age (years)	Diagnostic	Dyspnea RP-ILD	Skin*	CT	ELISA MDA5	IB MDA5	Other antibodies	Highest CK levels (IU/L)^∧^	Cancer	ICU	Lung pathology	LT	Exitus/ follow-up
1/F	April 2010 54	CADM	April 2010 Yes	—	NSIP	1.270	++	ANA (−) Ro52 (+) RF (+)	—	No	No	NA	No^#^	No June 2012

2/F	February 2005 57	DM	No	MH Panniculitis	Normal	1.637	+++	ANA 1/320	1856	Breast March 2007	No	—	No	No October 2012

3/M	June 2006 46	DM	February 2007 Yes	MH Ulcers	Ground glass	0.386	+	ANA (−) Ro52 (+)	4437	No	No	NA	No	No April 2012

4/F	November 1993 41	DM	April 2000 No	Panniculitis Calcinosis	Ground glass	1.343	+++	ANA (−)	304	No	No	NA	No	No October 2012

5^†^/F	March 2000 53	CADM	October 2000 Yes	Ulcers Panniculitis	Alveolar infiltrates	2.744	+++	ANA 1/640, Ro52 (+) RF (+)	—	No	November 2000	DAD^§^	Yes November 2000	Yes November 2000

6^†^/F	June 1992 28	CADM	August 1992 Yes	—	Alveolar infiltrates	1.220	++	ANA (−) Ro52 (+) RF (+)	—	No	September 1992	DAD^§^	No^‡^	Yes September 1992

7^†^/M	January 2000 69	CADM	May 2000 Yes	Ulcers	Ground glass	2.999	+++	ANA 1/160 U1RNP (+)	—	Lung March 2000	—	NA	No	Yes September 2000

8/F	February 1996 38	DM	No	—	Normal	1.340	+++	ANA 1/160 TIF1*γ* (+)	583	Ovarian July 1996	No	No	No	Yes December 1998

9/F	July 2004 30	CADM	No	Panniculitis Calcinosis	Normal	0.913	++	ANA (−)	—	No	No	No	No	No October 2012

10/M	May 1992 55	DM	No	Ulcers	Normal	2.251	+++	ANA 1/640 Ro52 (+)	136	Lung March 1996	—	No	No	Yes September 1997

11/M	June 2012 54	CADM	June 2012 Yes	—	Alveolar infiltrates	2.054	+++	ANA (−) Ro52 (+)	—	No	October 2012	DAD^§^	No^#^	Yes October 2012

12^†^/M	February 2000 46	CADM	February 2000 Yes	MH	Lung fibrosis	0.926	++	ANA (−)	—	No	November 2012	DAD^§^	Yes May 2000	Yes March 2004

13/F	March 2012 53	CADM	May 2012 Yes	MH	Alveolar infiltrates	2.456	+++	ANA (−) Ro52 (+)	—	No	No	NA	No^#^	Yes December 2012

14/F	July 2010 52	DM	No	Panniculitis	Normal	1.208	++	ANA (−)	550	No	No	No	No	No June 2012

ANA: antinuclear antibodies; CADM: clinically amyopathic dermatomyositis; DAD: diffuse alveolar damage; DM: dermatomyositis; F: female; ICU: intensive care unit; M: male; MH: mechanic's hands; NA: not available; NSIP: nonspecific interstitial pneumonia; RF: rheumatoid factor; RP-ILD: rapidly progressive interstitial lung disease. ^#^Proposed for lung transplantation (LT), but expired before it was available or improved and it was not necessary. ^†^Previously reported in [25]. ^§^Necropsy or lung explantation. ^‡^Not available in 1992. Patients 8 and 10 died from cancer and DM activity, respectively, and the remaining deceased patients died from acute respiratory failure. MDA5 value by ELISA is expressed in absorbance units. *All patients presented with classical skin manifestations (i.e., Gottron papules, heliotrope rash). The other skin manifestations reported in Table 1 are referred to nonclassic cutaneous involvement, and both (classic or not) are referred to the moment when dermatomyositis was diagnosed. ^∧^Creatine Kinase (CK). Normal value levels of CK (<195 IU/L).
